# Paddling through palpitations: when genes, myocardial inflammation and exercise collide—a case report of arrhythmogenic cardiomyopathy in a young competitive rower

**DOI:** 10.1093/ehjcr/ytaf442

**Published:** 2025-09-06

**Authors:** Boris Delpire, Olivier Ghekiere, Dagmara Dilling-Boer, Pieter Koopman, Guido Claessen

**Affiliations:** Jessa Hospital, Department of Cardiology, Hartcentrum, Stadsomvaart 11, 3500 Hasselt, Belgium; UHasselt, Faculty of Medicine and Life Sciences/LCRC, Agoralaan, 3590 Diepenbeek, Belgium; Department of Cardiovascular Sciences, KU Leuven, Herestraat 49, 3000 Leuven, Belgium; Department of Cardiovascular Diseases, UZ Leuven, Herestraat 49, 3000 Leuven, Belgium; UHasselt, Faculty of Medicine and Life Sciences/LCRC, Agoralaan, 3590 Diepenbeek, Belgium; Department of Radiology, Jessa Hospital, Stadsomvaart 11, 3500 Hasselt, Belgium; Jessa Hospital, Department of Cardiology, Hartcentrum, Stadsomvaart 11, 3500 Hasselt, Belgium; Jessa Hospital, Department of Cardiology, Hartcentrum, Stadsomvaart 11, 3500 Hasselt, Belgium; Jessa Hospital, Department of Cardiology, Hartcentrum, Stadsomvaart 11, 3500 Hasselt, Belgium; UHasselt, Faculty of Medicine and Life Sciences/LCRC, Agoralaan, 3590 Diepenbeek, Belgium; Department of Cardiovascular Sciences, KU Leuven, Herestraat 49, 3000 Leuven, Belgium

**Keywords:** Sports cardiology, Ventricular arrhythmias, Arrhythmogenic cardiomyopathy, Myocarditis, PKP2, Case report

## Abstract

**Background:**

Arrhythmogenic cardiomyopathy (ACM) is characterized by fibrofatty replacement of myocardium, predisposing to ventricular arrhythmias and sudden cardiac death. Arrhythmogenic cardiomyopathy is often linked to desmosomal gene mutations, particularly PKP2, which encodes plakophilin-2, a key structural protein in cardiac intercalated discs. In individuals with PKP2 mutations, exercise has been shown to accelerate disease progression.

**Case summary:**

A 22-year-old male semi-professional rower presented with palpitations, pre-syncope, and a history of presumed myocarditis with subepicardial fibrosis on cardiac magnetic resonance (CMR). Workup revealed anterior T-wave inversions on resting ECG and sustained monomorphic right ventricular (RV) outflow tract tachycardia, induced during exercise testing. Repeat CMR showed RV dysfunction and non-ischaemic RV and LV fibrosis with fibrofatty replacement. The patient met diagnostic criteria for biventricular ACM and underwent catheter ablation targeting the arrhythmic substrate. A multidisciplinary team carefully considered ICD therapy. However, due to the limited extent of the arrhythmic substrate, the exercise-induced nature of the ventricular tachycardia, and the successful ablation, ICD implantation was deferred at this stage. An ILR was implanted for continuous rhythm monitoring, with a low threshold for future ICD placement. High-intensity sports restriction, pharmacological therapy, and genetic counselling were initiated. Genetic testing identified a pathogenic PKP2 mutation.

**Discussion:**

This case highlights the complex interplay of genetic predisposition, myocardial inflammation, and exercise in ACM expression. The presumed myocarditis likely represented a ‘hot phase’ of ACM, accelerating structural cardiac changes. High-intensity exercise then acted as a ‘second hit,’ triggering phenotypic expression. Multidisciplinary evaluation combining rhythm monitoring, imaging, and genetic testing was key to diagnosis and management.

Learning points

- Ventricular tachycardia (VT) of right ventricular outflow tract (RVOT) origin is not invariably benign: although often presumed idiopathic, RVOT VT may be the initial presentation of arrhythmogenic cardiomyopathy (ACM). Multimodality assessment, which may include exercise testing, cardiac magnetic resonance imaging, and genetic testing, depending on the clinical context, is essential to exclude underlying ACM.
- Exercise as a ‘second hit’: high-intensity training may act as a ‘second hit’ in genetically predisposed athletes, such as those with PKP2 mutations, accelerating the onset and severity of ACM.
- Myocarditis or hot-phase of ACM: inflammatory episodes mimicking myocarditis may represent a hot-phase of ACM, contributing to myocardial injury and accelerating phenotypic expression, particularly in genetically susceptible athletes.

## Introduction

Arrhythmogenic cardiomyopathy (ACM) is a cardiac disease marked by progressive fibrofatty replacement of the myocardium, predisposing to ventricular arrhythmias and sudden cardiac death (SCD).^1,2^ Although originally described as a right ventricular disorder, ACM is now understood to include biventricular and left-dominant phenotypes, which can resemble other cardiomyopathies, complicating diagnosis.^3^ Most cases are associated with mutations in desmosomal genes, most notably PKP2, encoding plakophilin-2, a key component of intercalated discs.^4^ These mutations impair mechanical coupling and electrical conduction between cardiomyocytes, promoting arrhythmogenesis and adverse myocardial remodelling.

## Summary figure

**Figure:**
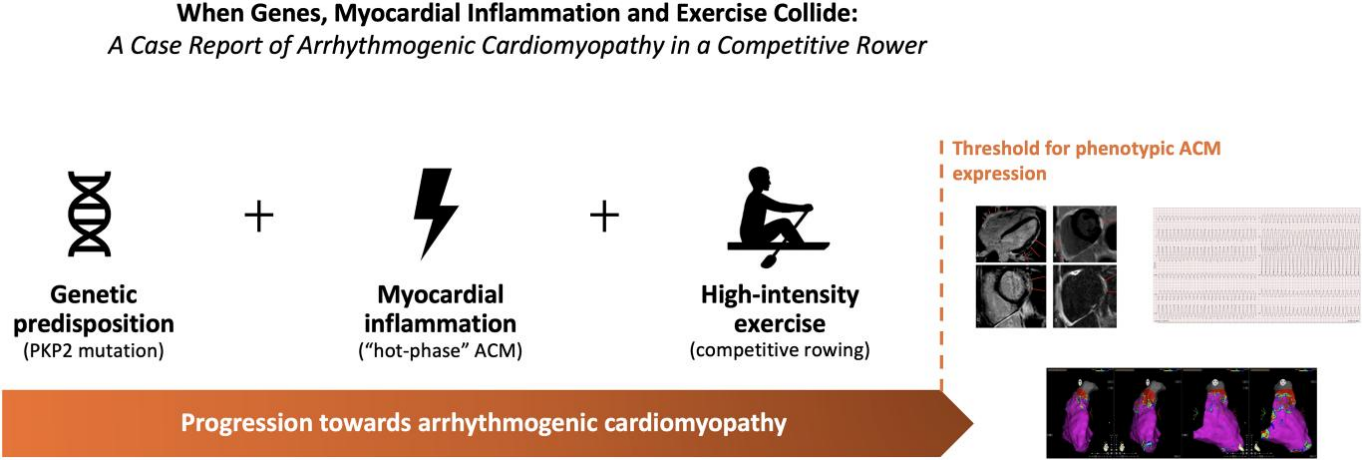


## Case presentation

A 22-year-old male endurance athlete and semi-professional rower presented to our sports cardiology clinic with recurrent, abrupt-onset palpitations, often accompanied by pre-syncope and nausea. During these episodes, his smartwatch recorded peak heart rates of up to 238 b.p.m.

His medical history includes a presumed myocarditis episode in 2020, which led to hospitalization in Italy; however, documentation from that event was unavailable. Upon his return to Belgium, a cardiac magnetic resonance (CMR) conducted at another facility showed subepicardial fibrosis in the basal and mid-ventricular region of the LV lateral wall, attributed to myocarditis, with a slightly reduced right ventricular ejection fraction (RVEF) of 50%. At that time, an ECG demonstrated T-wave inversion in V3, and laboratory results revealed a slightly elevated high-sensitivity troponin level of 43 ng/L. A 24-hour Holter monitor documented over 7000 premature ventricular beats (PVBs), which were attributed to myocarditis and managed conservatively.

His family history was notable for the SCD of his paternal grandfather at age 75. He trained approximately 15 h weekly at high intensity, primarily rowing. Physical examination was within normal limits, and he didn’t take any medications.

**Figure 1:**
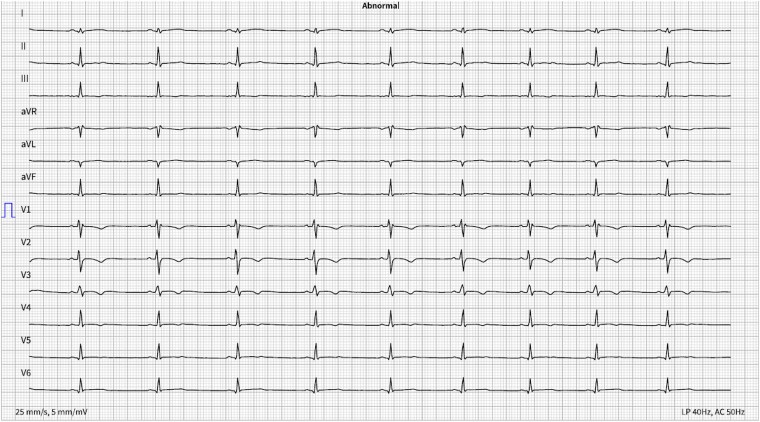
Resting 12-lead ECG showing T-wave inversions in leads V1 to V4.

A 12-lead resting ECG (*Figure 1*) showed T-wave inversions in the anterior leads (V1–V4), prompting further diagnostic evaluation. Transthoracic echocardiography revealed wall motion abnormalities in the posterior LV wall and mild hypokinesia in the mid-ventricular inferoposterior LV segment.

**Figure 2:**
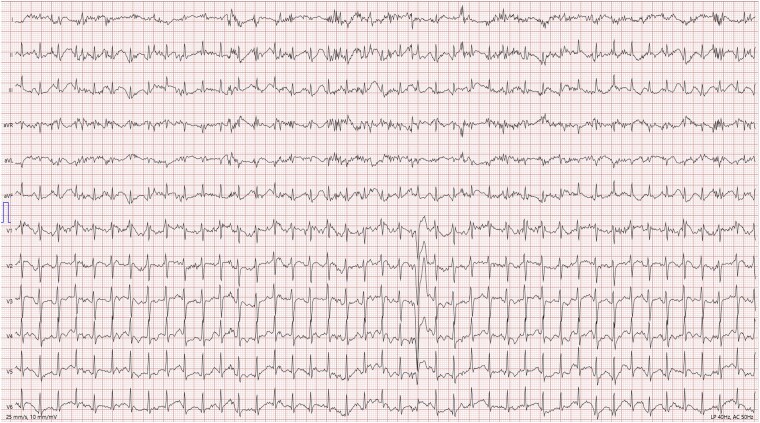
One of seven isolated PVBs with LBBB morphology and inferior axis observed during the exercise test.

**Figure 3:**
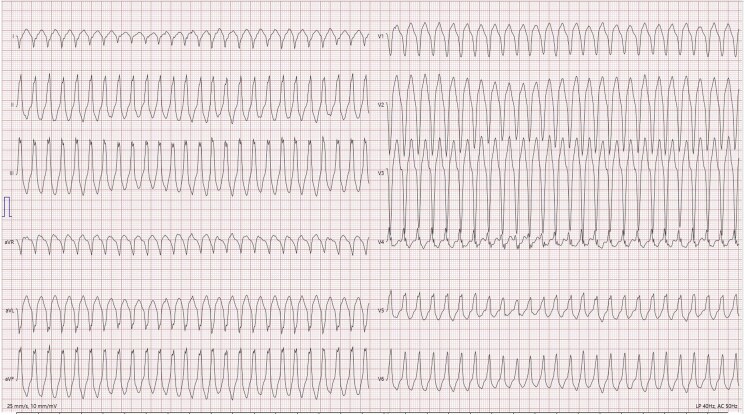
Rapid monomorphic ventricular tachycardia with LBBB morphology, transitioning in leads V5–V6 during the recovery phase of the exercise test.

The patient subsequently underwent a cycle ergometer exercise test with 12-lead ECG monitoring. During the test, seven isolated PVBs with left bundle branch block (LBBB) morphology with inferior axis were observed (*Figure 2*). One minute into the recovery phase, the patient experienced rapid monomorphic ventricular tachycardia (VT) with LBBB morphology, transitioning in leads V5–V6, as illustrated in *Figure 3*. The sustained VT resolved spontaneously during the recovery phase following the exercise test.

**Figure 4:**
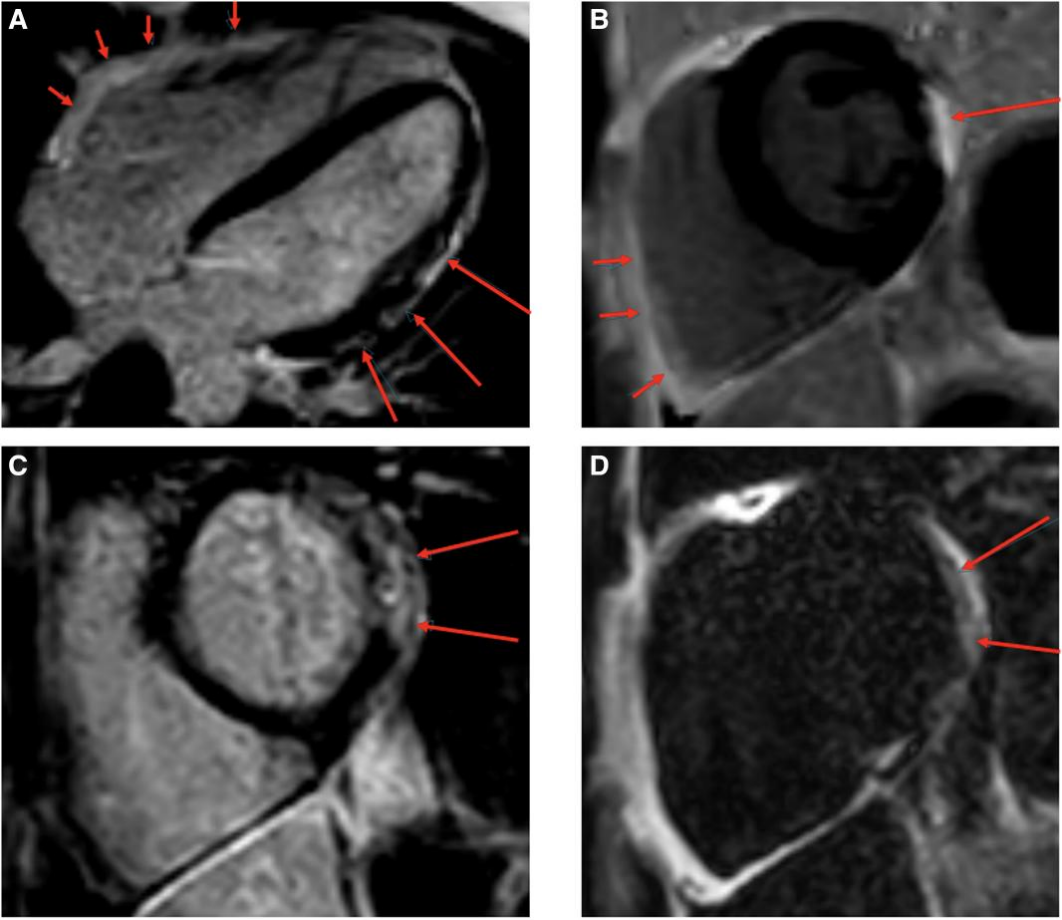
Cardiac magnetic resonance showing diffuse subepicardial late gadolinium enhancement in the lateral wall of the left ventricle (long arrows) and in the anterolateral right ventricular wall (short arrows) on four-chamber (*A*) and short-axis views (*B*). Short-axis Dixon LGE imaging with water-image (*C*) and corresponding-fat image (*D*) confirming non-ischaemic myocardial fibrofatty infiltration in the basal inferolateral wall of the left ventricle, meeting the criteria of biventricular ACM.

Biochemical analysis at our clinic revealed an elevated high-sensitivity troponin level of 156 ng/L following the episode of VT, which subsequently increased to 1280 ng/L. Thyroid function tests and C-reactive protein levels remained within normal ranges. CMR at our institution revealed RV dysfunction, with a reduced RVEF of 43% and regional hypokinesia in the anterolateral RV wall on cine sequences. Late gadolinium enhancement (LGE) imaging identified areas of non-ischaemic myocardial fibrosis in the anterolateral RV wall and the lateral LV wall, with fibrofatty infiltration (*Figure 4*). The patient fulfilled the diagnostic criteria for biventricular ACM, meeting three major criteria (global RV systolic dysfunction, fibrofatty myocardial replacement, and T-wave inversions in precordial leads V1–V4) and one minor criterion of sustained VT with right ventricular outflow tract (RVOT) pattern.^3^

**Figure 5:**
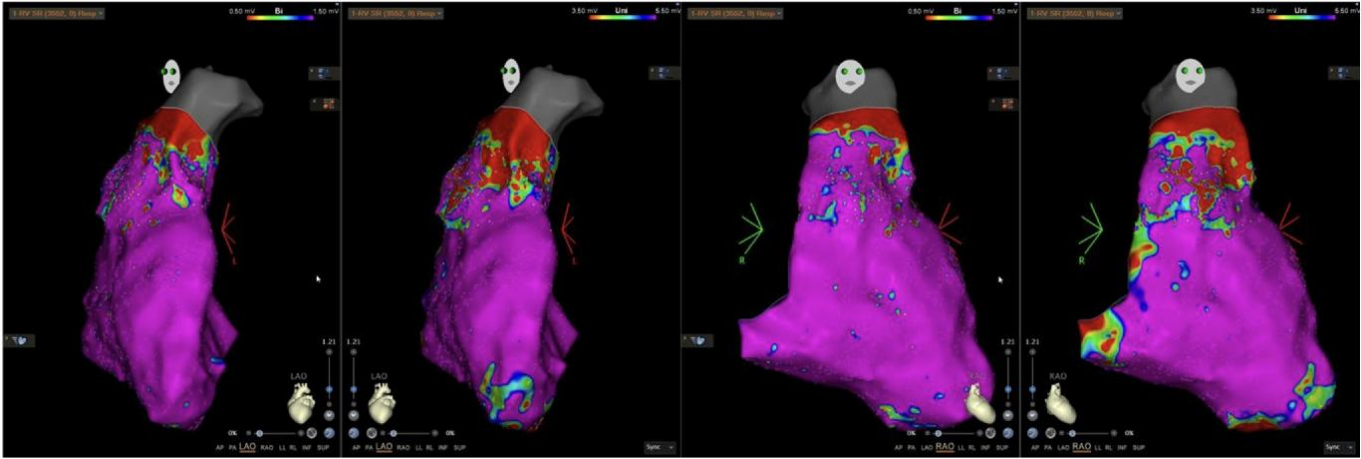
LAO view (two left images) and RAO view (two right images) showing patchy scar tissue in the anterolateral right ventricular outflow tract (RVOT).

An electrophysiology study demonstrated easily inducible episodes of both sustained and non-sustained VT. Electroanatomical mapping identified patchy scar tissue in the anterolateral RVOT (*Figure 5*), leading to catheter ablation targeting these scarred regions. An Ajmaline provocation test was negative. Subsequent exercise testing post-ablation did not induce further episodes of sustained or non-sustained VT.

Considering the patient’s young age, the limited arrhythmic substrate with successful ablation of the clinical VT, and the exercise-induced nature of the VT, our multidisciplinary team determined that immediate implantation of an ICD was not warranted. Instead, an ILR was implanted to enable continuous rhythm monitoring, and genetic testing was performed.

The patient was initiated on a low-dose beta-blocker (1.25 mg) and counselled to avoid competitive sports and moderate-to-high-intensity physical activities. These recommendations align with the European Society of Cardiology (ESC) guidelines, which highlight the association of such activities with accelerated disease progression, increased risk of ventricular arrhythmias, and adverse events in patients with ACM.^5^

Two weeks later, he began cardiovascular rehabilitation, during which exercise testing revealed frequent PVBs of a new morphology but no non-sustained VT. The ILR showed no VT events, and a 24-hour Holter recorded a PVB burden of 320 beats (0.4% of total beats) without complex arrhythmias.

Given the initially reduced RV function, a cardiopulmonary exercise test with echocardiography (CPET-echo) was conducted two months post-hospitalization to assess contractile reserve. The RV end-systolic pressure-area ratio (RVESPAR) was measured at 1.7, indicating a mildly reduced RV reserve.^6^ In light of these findings and the increased incidence of palpitations during rehabilitation, the beta-blocker dosage was adjusted to 1.25 mg twice daily. Consequently, rehabilitation was temporarily suspended, with a follow-up assessment scheduled in three months.

Genetic testing eventually identified a pathogenic PKP2 mutation (c.2489+1G>A) associated with ARVC, leading to a discussion on ICD implantation.^4,7,8^ Our team reassessed the patient with electrophysiology testing, which induced only one non-sustained VT episode of 8 beats. Given the absence of sustained or non-sustained VT on Holter monitoring, lack of inducible sustained arrhythmias during electrophysiology study, and limited arrhythmic substrate, ICD implantation was deferred. Genetic testing was advised for first-degree relatives. To improve symptom control, flecainide was added alongside the beta-blocker as part of the maintenance therapy. The patient’s long-term management includes regular rhythm monitoring and periodic imaging to assess ventricular function and evaluate ICD necessity.

## Discussion

This case report involves a young endurance athlete with symptoms suggestive of ventricular arrhythmia, ultimately diagnosed with biventricular ACM through ECG, cardiac imaging and genetic testing. His history included a suspected episode of myocarditis and around 15 h of weekly endurance training.

Research shows that physical exertion can exacerbate ACM or even trigger its onset.^9^ Several studies suggest intense endurance exercise serves as a ‘second hit’ for those vulnerable to ACM.^10,11^ Genetic testing revealed the patient was heterozygous for a pathogenic PKP2 mutation (c.2489+1G>A), classified with a pathogenicity score of 5/5 in the ClinVar database. This mutation is associated with ACM^4,7,8^ and is likely to lower the threshold for myocardial damage, with endurance exercise acting as the ‘second hit’. High-intensity exercise is therefore contraindicated in ACM patients with a pathogenic PKP2 mutation.^5^ The patient was advised to limit activities that raise his heart rate above 110 b.p.m., in line with ESC guidelines recommending low-intensity exercise below 55% of predicted maximum (198 b.p.m. for his age).^5^

Furthermore, the relationship between myocarditis and ACM progression, as noted by Martine *et al*., underscores how inflammatory episodes may accelerate structural cardiac changes, possibly leading to an earlier phenotype manifestation.^12^ A strong association between ACM and myocarditis has been proposed; alterations in desmosomal proteins are recognized as predisposing factors for various types of myocarditis.^13,14^ Acute myocarditis may reflect an active phase of ACM (so-called ‘hot-phase’), potentially accelerating disease progression and increasing arrhythmic risk.^15^

Importantly, VT originating from the RVOT (*Figure 3*) does not necessarily exclude ACM. Although RVOT-origin VT is often considered benign, thorough evaluation with exercise imaging, CMR, and genetic analysis can reveal ACM, as demonstrated in this case. It is important to emphasize that the abrupt onset of palpitations accompanied by malaise was a key clinical feature that raised suspicion and prompted further diagnostic evaluation.

In conclusion, the ACM phenotype manifests when myocardial damage exceeds a critical threshold, with genetic predisposition, inflammatory ‘hot-phase’ episodes, and exercise-induced stress collectively accelerating myocardial damage and disease progression in this specific case.

## Lead author biography


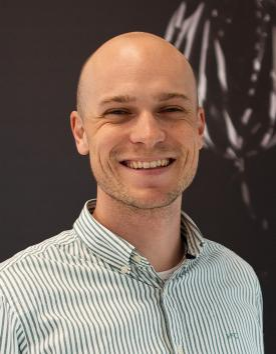


Dr Boris Delpire is an internal medicine resident at KU Leuven and a PhD candidate in sports cardiology under the supervision of Prof. Dr Guido Claessen and Prof. Dr Rik Willems. His research focuses on cardiac rhythm monitoring and arrhythmias in athletes. He is clinically active at Jessa Hospital in Hasselt as part of the sports cardiology team. He contributes to, amongst others, the Pro@Heart study, participating in the annual cardiovascular screening of hundreds of elite athletes.

## Author contributions

Boris Delpire (Visualization, Writing—original draft), Boris Delpire, Guido Claessen, Olivier Ghekiere, Pieter Koopman, Dagmara Dilling-Boer (Writing—review & editing).

**Consent:** Written informed consent was obtained from the patient for publication of this case report and any accompanying images, in accordance with COPE guidelines.

**Funding:** None.

## Data Availability

The data underlying this article will be shared on reasonable request to the corresponding author.
